# Effects of Concrete and Wood Building Environments on Pregnant Dams and Embryo-Fetal Development in Rats

**DOI:** 10.5487/TR.2009.25.4.209

**Published:** 2009-12-30

**Authors:** In-Sik Shin, Sung-Hwan Kim, Jeong-Hyeon Lim, Jong-Chan Lee, Na-Hyeong Park, Dong-Ho Shin, Changjong Moon, Sung-Ho Kim, Jong-Choon Kim

**Affiliations:** 17 Animal Medical Center, College of Veterinary Medicine, Chonnam National University, Gwangju, 500-757 Korea; 27 Department of Toxicology, College of Veterinary Medicine, Chonnam National University, Gwangju, 500-757 Korea

**Keywords:** Building materials, Concrete, Wood, Pregnancy, Embryo-fetal development, Rats

## Abstract

We have recently reported that the continuous exposure of rats to a concrete building environment under cool temperatures had adverse effects on general health parameters and embryo-fetal development. This study examined to compare the potential effects of concrete and wood building environments on pregnant dams and embryo-fetal development in rats. Groups of 10 mated females were exposed to polycarbonate (control), concrete, or wood cages from gestational days (GD) 0 to 20 under cool temperatures (11.9∼12.3°C). All the females underwent a caesarean section on GD 20, and their fetuses were examined for any morphological abnormalities. The temperatures in the cages were similar in all groups but the relative humidity in the concrete and wood groups were higher than in the control group. The concentration of volatile organic compounds in the wood group was higher than in the control group. In the concrete group, maternal effects manifested as an increase in the incidence of clinical signs, a lower body weight, and a decrease in the thymus and ovary weights. Developmental effects included increased post-implantation loss and decreased litter size. Infrared thermal analysis showed that the skin temperature of the rats in the concrete group was lower than that in the control group. In contrast, there were no exposure-related adverse effects on the maternal and developmental parameters in the wood group. Overall, the exposure of pregnant rats to a concrete building environment under cool temperatures has adverse effects on the clinical signs, body weight, skin temperature, organ weight, and embryo-fetal development. On the other hand, exposure to a wood building environment does not have any adverse effects in rats.

## INTRODUCTION

Housing provides human beings with a significant number of their physical and emotional needs. However, there is evidence that poor housing conditions contribute to increasing exposure to biological (e.g., allergens), chemical (e.g., volatile organic chemicals) and physical (e.g., thermal stress) hazards, which directly affect physiological and biochemical processes. Therefore, it is clear that the environmental conditions of home play an important role for individual and public health. In particular, the housing conditions such as ventilation, temperature, humidity and building materials are some of the main settings that affect human health (Krieger and Higgins, 2002).

Concrete is a hardened building material created by combining a chemically inert mineral aggregate (usually sand, gravel, or crushed stone), a binder (natural or synthetic cement), chemical additives, and water. Despite the considerable utility of concrete, environmental contamination and the potential health risk of concrete have recently attracted increasing attention. Previous studies demonstrated that cement and concrete had various adverse effects such as allergic contact dermatitis, skin irritation/corrosion, and genetic and respiratory diseases (Ruppersberger, 1995). We have also demonstrated that the continuous exposure of rats to a concrete building environment under cool temperatures had adverse effects on general health parameters and embryo-fetal development (Lee *et al.*, 2007, 2008). Recently, there has been growing concern regarding healthy housing using eco-friendly materials such as wood, soil, charcoal, etc (Kim, 2005). Wood has been used for thousands of years as one of the major eco-friendly building materials. In particular, pine tree is widely used to build houses in Korea (Kim, 2005).

This study examined the potential adverse/beneficial effects of concrete and wood building environments on pregnant dams and embryo-fetal development after maternal exposure during the entire period of pregnancy in Sprague-Dawley rats.

## MATERIALS AND METHODS

***Rat housing system.*** Each housing system consisted of 6 individual cages constructed from polycarbonate, concrete, or wood (pine tree). The individual cage sizes of the concrete and wood housing systems were identical to the commercial polycarbonate cage (240 W × 390 L × 175 H mm; Gyeryong Co, Daejeon, Korea) except for the cage thickness as shown in Fig. 1. Polycarbonate was used for comparison. In order to maintain a limited indoor-air circulation similar to a human house, the housing systems were covered with thin polycarbonate lids (4 mm) to allow a 5 cm opening in the wall. Although there were some differences in the housing conditions such as temperature, humidity, ventilation, and construction finish material between human and rat housing systems, the experimental housing system was designed based on the usual housing conditions for humans as well as for the potential exposure routes (direct dermal contact and inhalation) for building materials.

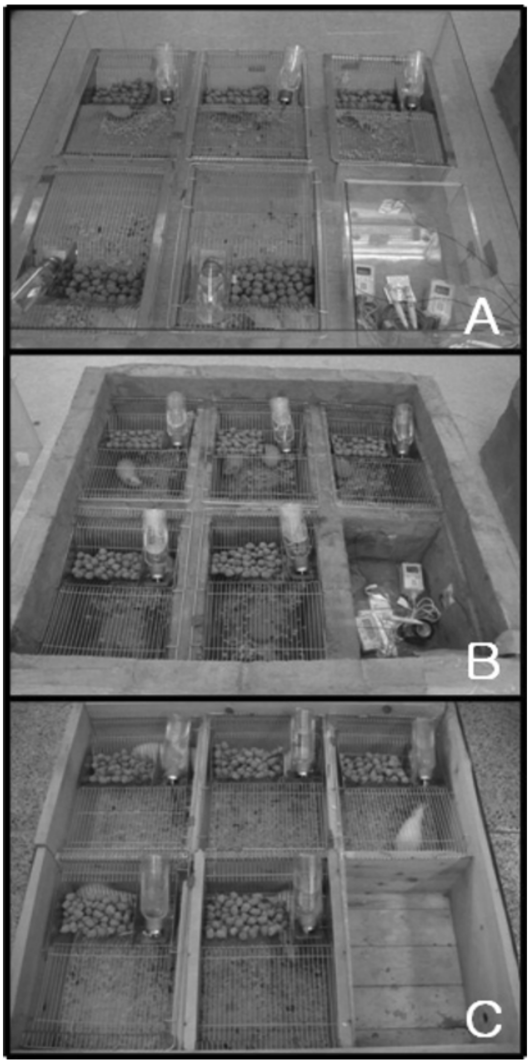

**Fig. 1.** Photographs of the polycarbonate (A), concrete (B), and wood (C) housing systems.

***Animal husbandry and maintenance.*** Male and nulliparous female Sprague Dawley rats aged 10 weeks were obtained from a specific pathogen-free colony at Orient Bio Inc. (Seoul, Korea) and were used after allowing 1 week for quarantine and acclimatization. This strain was chosen because it is most commonly used in reproductive and developmental toxicity studies, and historical control data is available. The animals were housed in a room under ambient temperature and humidity encountered in early winter with artificial lighting from 08:00 to 20:00. For mating, two females were placed overnight into a polycarbonate cage with a male rat. Successful mating was confirmed by the presence of sperm in the vaginal smear, and the following 24 h was designated as day 0 of gestation (GD 0). The mated females were housed two per cage in polycarbonate, concrete, or wood cages with stainless steel wire lids, and were given tap water and commercial rodent chow (Samyang Feed Co., Weonju, Korea) ad libitum. In order to exclude the potential adverse effects of a high-humidity environment due to urine, feces, and tapwater within the cages, the same amount (2 g/rat/day) of wood bedding sufficient to absorb the urine and tapwater was supplied in the morning and replaced daily. The Institutional Animal Care and Use Committee of Chonnam National University approved the study protocols and the animals were maintained in accordance with the Guide for the Care and Use of Laboratory Animals (NRC, 1996).

***Measurement of temperature and relative humidity in each housing system.*** The temperature and relative humidity in the internal air of housing systems were measured daily using a T-type thermocouple (OMEGA, USA).

***Measurement of total volatile organic compounds (VOCs) in each housing systems.*** The concentration of total VOCs within each housing system was measured immediately before the beginning of exposure and once a week during the exposure period using a Toxic-1000 Portable Vapor Analyzer (USA). The VOCs in the internal air of cages covered with thin polycarbonate lids were measured over a 1 hour period.

***Measurement of skin temperature.*** The skin temperature of the rats in each housing system was measured immediately before the exposure and on GD 1, 7, 14, and 20 using an infrared thermal imaging system (Thermo Tracer TH5100, NEC, Japan).

***Observation of dams.*** All the pregnant females were examined daily throughout the gestation period for mortality, general appearance, and locomotor activity by cage-side observation. The maternal body weights were measured on GD 0, 3, 6, 9, 12, 15, and 20, and the level of individual food consumption was determined on GD 0, 3, 6, 9, 12, 15, and 19. *The amounts of food* were *calculated before* they were *supplied to each cage*, and their *remnants* were measured on the next day in order to *calculate* the differences which were regarded as daily food consumption (g/rat/day). At the scheduled termination day (GD 20), all the pregnant females were euthanized by ether inhalation and exsanguination from the aorta. A complete gross postmortem examination was then performed. The absolute and relative (organ-to-body weight ratio) weights of the lung, adrenal glands, liver, spleen, kidneys, thymus, heart, and ovaries were measured in all the survivors.

***Post-mortem examination.*** The ovaries and uterus of each female were removed and examined for the number of corpora lutea, and the status of all implantation sites, i.e., live and dead fetuses, early and late resorptions, and total implantations. Uteri with no evidence of implantation were stained with a 2% sodium hydroxide solution to determine the presence of early resorption sites (Yamada *et al*., 1985). The rat was considered not pregnant if there were no stained implantation sites present. Resorption was classified as “early” when only a resorption site resembling a dark brown blood clot and with no embryonic tissue was visible, and was considered “late” when both the placental and embryonic tissues were visible at the post mortem examination. All live fetuses were weighed individually, sexed, and examined for any morphological abnormalities, including a cleft palate. The alternate fetuses were selected for either a skeletal or visceral examination. Half of the live fetuses from each litter were fixed in 5% formalin solution, eviscerated, and then processed for skeletal staining with alizarin red S using a modification of the method reported by Dawson (1926) for a subsequent skeletal examination. The other half was preserved in Bouin’s solution and examined for any changes in the internal soft tissue using a freehand razorsectioning technique (Wilson, 1965) and Nishimura’s method (1974). All fetal morphological alterations observed were classified as either developmental malformations or variations. A malformation was defined as a permanent structural change that is likely to adversely affect the rat’s survival or health (Chahoud *et al*., 1999). The term “variation” was defined as a change that occurs within a normal population and is unlikely to adversely affect the survival or health. The terminology suggested in an internationally developed glossary of terms was used to classify the structural developmental abnormalities in common laboratory mammals (Wise *et al*., 1997).

***Statistical analysis.*** The unit for statistical measurement was a pregnant dam or a litter (Weil, 1970). Statistical analysis of the experimental and control values was performed using a Student’s t-test. The gender ratio and the proportion of the litters with malformations and developmental variations were compared using a chi-square test and Fisher’s exact probability test (Fisher, 1970). The statistical analyses were performed using GraphPad InStat (Graph-Pad Software Inc. San Diego, CA) software. The significance of the differences between the groups was estimated at the probability levels of 1 and 5%.

## RESULTS

***Temperature, relative humidity, and tVOCs.*** As shown in Table 1, the temperatures in the cages were similar in all groups but the relative humidity in the concrete and wood cages were significantly higher than that in the control group. The concentration of tVOCs in the wood cage was significantly higher than that in the control group during the study period.

***Maternal findings.*** There were no exposure-related deaths in any of the animals placed in the polycarbonate, concrete, and wood building environments during the pregnancy period (data not shown). However, exposure-related clinical signs were observed in the polycarbonate and concrete groups, which included decreased locomotor activity, soft stools, a stained/wet perineum, and a loss of fur. There were 2 rats in the control group each showing decreased locomotor activity and soft stools, and 1 case with a stained/wet perineum. In the concrete group, there were 4 cases showing decreased

**Table 1.** Temperature, relative humidity, and the concentration of total volatile organic compounds (tVOC) in the rat housing systems

| Items | Polycarbonate | Concrete | Wood |
|---|---|---|---|
| Temperature (°C) | 12.3 ± 1.99[a)] | 11.9 ± 1.97 | 12.2 ± 1.73 |
| Relative humidity (%) | 71.8 ± 4.69 | 76.5 ± 7.58* | 79.6 ± 4.66** |
| tVOC before study (ppm) | 0 | 1.8 | 5.7 |
| tVOC during study (ppm) | 32.3 ± 3.54 | 26.6 ± 4.01 | 54.0 ± 13.5**,† |

[a)]Values are presented as mean ± SD.
*,**Indicate a significant difference at $P < 0.05$ and $P < 0.01$ levels compared with the polycarbonate group, respectively.
†Indicates a significant difference at $P < 0.05$ level compared with the concrete group.

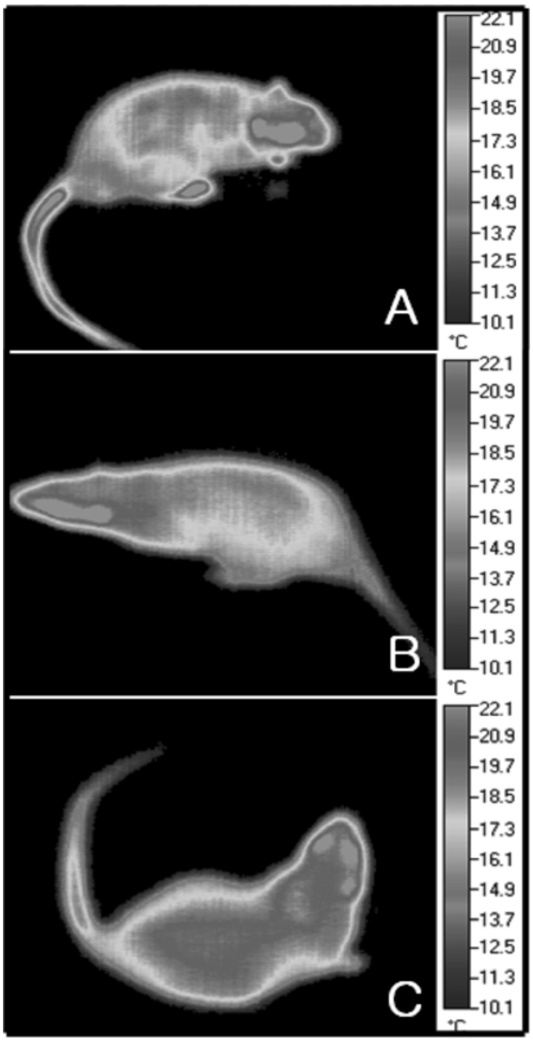

**Fig. 2.** Representative photographs of the skin temperature of the rats raised for 14 days in the polycarbonate (A), concrete (B), and wood (C) cages.

locomotor activity, 2 cases with soft stools, a stained/wet perineum and a loss of fur, and 1 case of diarrhea. In contrast, there was only 1 case with a stained/wet perineum in the wood group. As shown in Fig. 2, the skin temperature in the concrete group was lower from gestational days 7 to 20, particularly in the parts of limbs and tail, than those in the polycarbonate and wood groups.

Table 2 shows the changes in body weight in each group during the study period. The maternal body weights at GD 15 and 20 and the weight gain during the pregnancy in the concrete group were significantly lower than the control group. In contrast, the maternal body weight and body weight gain during pregnancy in the wood group were similar to those in the control group. The level of food consumption showed a tendency to decrease in the concrete group but there was no statistically significantly difference between the groups during the pregnancy period (data not shown).

There were no exposure-related gross findings in any of the groups at the scheduled necropsy (data not shown). As shown in Table 3, the absolute and relative weights of the thymus and the absolute weight of the ovaries were significantly lighter in the concrete group than in the control group. In contrast, the absolute weights of the thymus and ovaries in the wood group were significantly higher than in the concrete group.

***Embryo-fetal findings.*** Table 4 summarizes the reproductive findings of the pregnant rats exposed to the polycarbonate, concrete, and wood building environments during pregnancy. The overall pregnancy rates were similar in all groups, ranging from 90~100%. No totally resorbed litters were found in any group. The number of corpora lutea, implantations, pre-implantation loss rates, gender ratio and body weight of live fetuses, and the placental weight were similar in all groups. However, the rate of post-implantation loss in the concrete group was significantly higher than that in the control group. The litter size in the concrete group was significantly lower than that in the control group. In contrast, no adverse effects on the rate of implantation loss and litter size were observed in the wood group.

Table 5 shows the types and incidence of external, visceral, and skeletal malformations as well as variations observed in the F1 fetuses. Although there were

**Table 2.** Body weight of the pregnant rats housed in polycarbonate, concrete, and wood cages during pregnancy

| Items | Polycarbonate | Concrete | Wood |
|---|---|---|---|
| No. of mated females | 10 | 10 | 10 |
| No. of pregnant females | 9 | 9 | 10 |
| Gestational day 0 | 251.7 ± 21.66[a)] | 246.3 ± 18.29 | 247.3 ± 11.36 |
| Gestational day 3 | 260.4 ± 13.56 | 253.0 ± 18.38 | 260.0 ± 13.87 |
| Gestational day 6 | 272.3 ± 15.99 | 257.5 ± 16.49 | 271.8 ± 12.44 |
| Gestational day 9 | 280.9 ± 17.02 | 265.9 ± 16.63 | 278.8 ± 12.44 |
| Gestational day 12 | 297.1 ± 16.11 | 283.8 ± 15.91 | 293.6 ± 14.30 |
| Gestational day 15 | 311.9 ± 18.90 | 288.2 ± 20.12* | 308.9 ± 18.04† |
| Gestational day 20 | 373.6 ± 29.35 | 340.8 ± 30.10* | 367.0 ± 26.14 |
| Weight gain during pregnancy | 121.9 ± 19.03 | 94.4 ± 32.64* | 119.7 ± 19.34 |
| Corrected body weight[b)] | 297.4 ± 21.07 | 280.7 ± 16.34 | 293.5 ± 13.11 |
| Gravid uterine weight | 76.2 ± 20.06 | 60.1 ± 24.32 | 73.5 ± 17.83 |

[a)] Values are presented as mean ± SD (g).
[b)]Corrected body weight = Body weight on gestational day 20 - gravid uterine weight.
*Indicates a significant difference at $P < 0.05$ level compared with the polycarbonate group.
†Indicates a significant difference at $P < 0.05$ level compared with the concrete group.

**Table 3.** Organ weights of the pregnant rats housed in polycarbonate, concrete, and wood cages during pregnancy

| Items | Polycarbonate | Concrete | Wood |
|---|---|---|---|
| No. of pregnant rats | 9 | 9 | 10 |
| Body weight at term (g) | 297.4 ± 21.07[a)] | 280.7 ± 16.34 | 293.5 ± 13.11 |
| Lung (g) | 1.29 ± 0.212 | 1.21 ± 0.135 | 1.25 ± 0.079 |
| per body weight (%) | 0.43 ± 0.043 | 0.43 ± 0.042 | 0.43 ± 0.030 |
| Adrenal glands (g) | 0.073 ± 0.0167 | 0.078 ± 0.0104 | 0.075 ± 0.0114 |
| per body weight (%) | 0.025 ± 0.0059 | 0.028 ± 0.0040 | 0.026 ± 0.0038 |
| Liver (g) | 15.11 ± 1.127 | 15.11 ± 1.127 | 14.74 ± 1.150 |
| per body weight (%) | 5.10 ± 0.360 | 5.01 ± 0.521 | 5.02 ± 0.288 |
| Spleen (g) | 0.68 ± 0.122 | 0.60 ± 0.057 | 0.65 ± 0.082 |
| per body weight (%) | 0.23 ± 0.048 | 0.21 ± 0.016 | 0.22 ± 0.027 |
| Kidneys (g) | 1.93 ± 0.146 | 1.84 ± 0.185 | 1.98 ± 0.145 |
| per body weight (%) | 0.65 ± 0.032 | 0.66 ± 0.041 | 0.67 ± 0.034 |
| Thymus (g) | 0.29 ± 0.066 | 0.21 ± 0.030** | 0.26 ± 0.061† |
| per body weight (%) | 0.10 ± 0.019 | 0.08 ± 0.014* | 0.09 ± 0.022 |
| Heart (g) | 1.03 ± 0.112 | 1.05 ± 0.087 | 0.98 ± 0.084 |
| per body weight (%) | 0.35 ± 0.032 | 0.37 ± 0.037 | 0.33 ± 0.026 |
| Ovaries (g) | 0.122 ± 0.0221 | 0.101 ± 0.0107* | 0.129 ± 0.0310† |
| per body weight (%) | 0.041 ± 0.0078 | 0.036 ± 0.0042 | 0.044 ± 0.0112 |

[a)] Values are presented as mean ± SD.
*,**Indicate a significant difference at $P < 0.05$ and $P < 0.01$ levels compared with the polycarbonate group, respectively.
†Indicates a significant difference at $P < 0.05$ level compared with the concrete group.

some types of visceral variations including a misshapen thymus, hemorrhagic kidney, umbilical hernia, and dilated ureter and skeletal variations including supernumerary ribs and dumbbell/bipartite ossification of the vertebral centrum, there were no significant differences in the number of fetuses with developmental variations or in the number of litters with affected fetuses among the groups.

## DISCUSSION

The present study examined the potential adverse/beneficial effects of concrete and wood building environments on pregnant dams and embryo-fetal development in Sprague Dawley rats. Significant effects on pregnant dams and embryo-fetal development were observed in the concrete group, but not in the wood group.

The relative humidity in the concrete and wood cages and the concentration of tVOCs in the wood cage were higher than those in the polycarbonate cage, respectively. However, it is believed that they were not of toxicological significance, because the increments were minimal and there were no adverse effects on both the pregnant dams and embryo-fetal development found in the wood group. The increased tVOCs observed in the

**Table 4.** Caesarean section and fetal examination data of the pregnant rats housed in polycarbonate, concrete, and wood cages during pregnancy

| Items | Polycarbonate | Concrete | Wood |
|---|---|---|---|
| No. of mated females | 10 | 10 | 10 |
| No. of pregnant females | 9 | 9 | 10 |
| No. of dead females | 0 | 0 | 0 |
| No. of litters totally resorbed | 0 | 0 | 0 |
| Corpora lutea | 15.2 ± 1.39[a)] | 14.1 ± 1.45 | 15.8 ± 1.32 |
| Implantation sites | 14.0 ± 2.45 | 12.2 ± 2.68 | 13.8 ± 2.97 |
| Pre-implantation loss (%) | 8.4 ± 12.81 | 14.1 ± 12.80 | 12.8 ± 15.87 |
| Fetal deaths | 0.7 ± 0.87 | 2.1 ± 1.96 | 1.0 ± 1.33 |
| Post-implantation loss (%) | 4.3 ± 5.48 | 16.75 ± 16.12* | 8.0 ± 10.89 |
| Litter size | 13.3 ± 2.12 | 10.1 ± 2.85* | 12.8 ± 3.43 |
| Male/female | 64/56 | 45/46 | 67/59 |
| Gender ratio | 1.14 | 0.98 | 1.14 |
| Fetal weight (g): Male | 3.7 ± 0.66 | 3.8 ± 0.38 | 3.8 ± 0.33 |
| Female | 3.4 ± 0.40 | 3.5 ± 0.37 | 3.7 ± 0.32 |
| Placental weight (g) | 0.43 ± 0.056 | 0.50 ± 0.085 | 0.48 ± 0.057 |

[a)]Values are presented as mean ± SD.
*Indicates a significant difference at $P < 0.05$ level compared with the polycarbonate group.

**Table 5.** External, visceral, and skeletal findings in fetuses of pregnant rats housed in polycarbonate, concrete, and wood cages during pregnancy

| Items | Polycarbonate | Concrete | Wood |
|---|---|---|---|
| *External examination* | | | |
| Litters examined | 9 | 9 | 10 |
| Fetuses examined | 120 | 91 | 128 |
| Fetuses with external anomalies | 0 | 0 | 0 |
| *Visceral examination* | | | |
| Litters examined | 9 | 9 | 10 |
| Fetuses examined | 57 | 43 | 62 |
| Fetuses with visceral malformation | 0 | 0 | 0 |
| Litters with visceral variations (%) | 2(22.2) | 4(44.4) | 3(30.0) |
| Fetuses with visceral variations (%) | 3(5.3) | 5(11.6) | 4(6.5) |
| *Skeletal examination* | | | |
| Litters examined | 9 | 9 | 10 |
| Fetuses examined | 63 | 48 | 66 |
| Fetuses with skeletal malformations | 0 | 0 | 0 |
| Litters with skeletal variations (%) | 9(100) | 9(100) | 9(90.0) |
| Fetuses with skeletal variations (%) | 41(65.1) | 26(54.2) | 37(56.1) |

wood cages during the study period might be due to the release of biogenic VOCs from urine and feces of rats absorbed in the wood materials.

Infrared thermal analysis showed that the skin temperature of the rats in the concrete group was lower than in the polycarbonate and wood groups. It should be noted that body temperature might fall as a result of heat loss from conduction, radiation, evaporation, convection, and respiration. The thermal conductivity of a material is dependent on its temperature, density, and moisture content, and generally decreases when the porosity of a material increases (Sastry *et al.*, 2004). Because the thermal conductivity (0.04~0.4 W/m/°C) of wood, which has a high porous structure, is much lower than that (1.7 W/m/°C) of concrete (Callister, 2003), it is believed that the lower skin temperature observed in the concrete group under cool temperature was mainly due to the relatively higher thermal conductivity. On the other hand, our previous study demonstrated that the concrete building environment in the summer did not have any exposure-related adverse effects on the general health parameters and skin temperature of the rats (Lee *et al.*, 2007).

Maternal effects observed in the concrete group manifested as an increase in the incidence of abnormal clinical signs including decreased locomotor activity, soft stools, a stained/wet perineum, and diarrhea, a decrease in the body weight gain and a decrease in the weights of thymus and ovaries. These findings are not known to occur in normal Sprague Dawley rats (Yang *et al.*, 2007) and the incidence and severity of these findings were much higher than controls. Therefore, it is believed that the maternal effects observed in the concrete group were probably due to heat loss as a result of exposure to the cool environment. It is well known that chemical, biological, physical, and social stresses can have adverse effects on humans and experimental animals (Rees, 1976). Previous studies have reported that the changes in the environmental temperature also induce various stress responses, including physiological and psychological alterations (McDonald *et al.*, 1996).

Developmental effects found in the concrete group included an increase in the post-implantation loss and a decrease in the litter size. These findings indicate that continuous exposure to cool temperatures in concrete building environment is embryotoxic to pregnant rats. Although a statistically significant difference was not detected, the increased number of fetal deaths observed in the concrete group is also considered to be an exposure-related effect. This is because there were a much higher (3 times) number of fetal deaths in the concrete group than in the control group, and the value was beyond the limits of the historical control rat fetuses (Kim *et al.*, 2001; MARTA, 1997; Morita *et al.*, 1987). We have recently demonstrated that continuous exposure of the rats to the concrete building environment under cool temperature had significant adverse effects on general health and embryo-fetal development in rats (Lee *et al.*, 2007, 2008).

In contrast, there were no exposure-related effects on the maternal and developmental parameters in the wood group, even with a similar low temperature. The results suggest that the wood building environment can efficiently prevent the potential adverse effects of cool temperatures on the pregnant dams and embryo-fetal development compared with the concrete building environment.

In conclusion, the continuous exposure of a concrete building environment to rats under cool temperature had some adverse effects on the clinical signs, body weight, skin temperature, organ weight, and embryo-fetal development. In contrast, the exposure of wood building environment did not have any exposure-related effects on pregnant dams or on embryo-fetal development in rats. Although there are some differences in the housing conditions such as temperature, humidity, ventilation, and construction finish material between human and rat housing systems, these results are expected to provide some information relevant to the safety assessment of concrete and wood building environments in pregnant women exposed to cold weather.

## ACKNOWLEDGMENT

This work was supported by the Grant of the Korean Ministry of Education, Science and Technology (The Regional Core Research Program/Bio-housing Research Institute). This work was also supported by the Grant of the Animal Medical Center, College of Veterinary Medicine, Chonnam National University.
